# Lifestyle and eating habits changes among adults during COVID-19 era in Egypt: a population-based study

**DOI:** 10.1186/s40795-024-00852-y

**Published:** 2024-03-19

**Authors:** Hebat-Allah Mohammed Salah Gabal, Ayat F. Manzour

**Affiliations:** Community, Environmental and Occupational Medicine Department, Faculty of Medicine, Ain Shams University, Cairo, Egypt

**Keywords:** Eating habits, Physical activity, Pandemic, Body Mass Index (BMI)

## Abstract

**Background:**

The 2019 recent Coronavirus is without a doubt one of the most complicated viruses to ever pose a threat to humanity. Numerous viral containment strategies forced sedentary behaviors and dietary changes that would–otherwise- increase the chances of acquiring non-communicable diseases.

**Objectives:**

The objectives of the current study are to identify any changes in eating behaviors through the Mediterranean Diet Adherence in a sample of Egyptians throughout the COVID-19 era.

**Methods:**

A cross-sectional study was done on a sample of 205 Egyptians by an online self-administered questionnaire. The questionnaire included socio-demographic factors, self-reported weights and heights, a validated Arabic form of the well-known International Physical Activity Questionnaire Short Form (IPAQ-SF), a validated Arabic version of 14-items Mediterranean Diet Adherence Screener (MEDAS), in addition to a section assessing dietetic changes. The data was then analyzed using the SPSS version 20 (Statistical Package for Social Sciences).

**Results:**

The majority of the study sample were females (74.6%); had a high level of education (93.2%); and about 75% were married. Most of the participants were non-smokers, with around a 7% increased frequency of smoking after the COVID-19 pandemic. Fast food consumption was also reported by a major percentage of study participants (60%). Low Mediterranean Diet Adherence was found in 52.7%. Moreover, Physical Activity (PA) decreased to 61%. Moreover, there was a statistically significant increase seen in the participants’ BMI as well as the number of sleeping hours (*p* = 0.001 and 0.043 respectively) after the pandemic. Both changed hunger sensation and any changes in physical activity were significantly associated with increased BMI (*p* < 0.001).

**Conclusion and recommendations:**

A substantial proportion of the participants showed unhealthy changes in their dietary habits as well as physical activity. Consequently, this calls for urgent public health policies and interventions to guard against the consequences of such unhealthy behaviors.

## Introduction

By the end of 2019, the world was hit with the Coronavirus (COVID-19) pandemic, a serious pandemic that prompted the adoption of forced public health measures to stop the virus from spreading. Following the Egyptian Ministry of Health’s declaration of the first case in the country, the World Health Organization (WHO) was then alerted of the situation [1]. 

It was not long after this that the Higher Committee to Combat Coronavirus, which was chaired over by the Prime Minister, took the responsibility of carrying out the national policy and institutional response [2]. 

As a result, in March 2020, corresponding at the beginning of the Coronavirus spread, the Egyptian government instigated a national lockdown and increased social isolation measures to stop the spread of COVID-19. On March 15th, 2020, Egyptian authorities began their efforts by closing schools and universities as preventive measures. Subsequently, lockdown was imposed, and more severe containment measures were decided [3]. 

As COVID-19’s second wave (which was near the end of 2020) approached, various containment procedures were also carried out. On November 12th, the Egyptian government declared the setting of new closing times to be imposed on public retail outlets, malls, restaurants, as well as cafés that would start on December 1st, 2020 [2]. 

Therefore, Egypt started to experience a second wave of the outbreak towards the end of the year, with daily reported cases reaching a peak of 1,400 people. Anticipated efforts to stop a second wave of illness were also implemented in January 2021. Following that, the Egyptian authorities implemented anti-COVID-19 preventative measures to control further waves of viral propagation [2, 4]. 

Generally speaking, whether partial or complete, any lockdown would have an impact on people’s social lives as all necessary public spaces, companies, and services have to be shut down. As for during the COVID-19 lockdown, everyone was forced to stay indoors and only leave their homes for essential requirements. As a result, the majority of people were either required to work remotely or even experienced temporary unemployment. Students were also forced to rely on digital learning. It was also seen that the population’s habits and lifestyles underwent a fast and dramatic transition, with a sharp decline in all forms of sociability. Thus, the physical isolation may have also had a significant negative impact on citizens’ lives, particularly on their eating patterns and daily routines [5]. 

Based on this lockdown, nutritional habits changed, especially because of altered food access. The people’s Physical Activity (PA) was reduced as a result of stopping daily travel and stopping trips to the grocery store, which are typically the only out-of-home activities for most people [5, 6]. 

Since the beginning of COVID-19, the studies that have been done have shown an increase in the presence of food insecurity as well as difficulties with access to food for the already-food-insecure populations. In these conducted studies, people who were food-insecure felt more worried about food access and expressed difficulties in finding the different types of foods they needed or wanted; in having sufficient food through the different food assistance programs as well as the emergency food organizations; and in not being able to manage to pay for food stock for two weeks in advance as recommended [7, 8]. 

For example, in Iran, the households were generally affected by food insecurity during COVID-19. This showed that 61% of the participants faced this feeling of food insecurity. That is not to mention that other economic, psychological, as well as other human aspects of exposure had the highest impact on the food insecurity during the first COVID-19 lockdown [9]. 

In another study that was conducted in three countries (Denmark, Germany, and Slovenia), a different pattern of alteration in food access was measured. A decrease in the frequency of shopping was significantly related to a decline in the act of consuming fresh food. Additionally, this decrease in the frequency of shopping was substantially correlated with an increase in the use of frozen food as well as canned food. In other words, this indicates that some consumers may have partially substituted using frozen food and canned food for alternative fresh food. Moreover, this decrease in the frequency of shopping was also greatly linked with an increase in eating sweet snacks.” [10]. 

That being said, one of the main risk causes of chronic diseases (non-communicable diseases) that can be modified is nutrition (diet and lifestyle factors, such as physical activity), which has been shown to raise the possibility of more severe COVID-19 consequences [11]. Based on this, the Mediterranean Diet (MD) is rich in micronutrients (e.g.: selenium, zinc, some vitamins etc…). Hence, when there is an inadequate intake of the MD, this exposes people to specific oxidative damage; thus, increasing the likelihood of being infected with COVID-19 [12, 13]. A reduced intake of the MD may also lead to the deficiency of micronutrients which may have a role in both obesity and impaired immune response) [14, 15]. 

It has been demonstrated that stress and emotional eating are related. Stressed individuals generally crave more high-fat and high-sugar food items [16]. An overwhelming need to eat is an emotional (i.e. having a strong wish to eat), or behavioral (i.e. searching for food), or cognitive (i.e. contemplating eating), or physiological (i.e. salivating) phenomenon that falls under the category of “food craving.” Cravings for carbohydrates promote the synthesis of serotonin, which boosts mood in proportion to a food’s glycemic index [17–19]. Stress from any quarantine also affects sleep, which raises stress levels and causes people to eat more, creating a hazardous feedback loop [20]. 

Moreover, extended periods of inactivity are also linked to adverse health effects [21]. Governments restricted most outside exercise as well as social activities (including heading to the gym) during quarantine, which decreased PA [22]. As for the effect of a lockdown and its precautionary measurement on lifestyle and eating habits, it can have a long-lasting impact on a person’s way of life and dietary habits. On the other hand, providing foods that are good sources of nutrients that improve the body’s immunity; planning the specific mealtimes and quantities; and scheduling a cut-off time for eating at night would aid in preventing the negative effects of any lockdown [23]. 

To prove this, various studies that have been done examined the impact of the COVID-19 pandemic on people’s eating habits as well as lifestyle changes. These studies included one which was done on an Italian population aged ≥ 12 years. Its findings revealed that the pandemic affected the perception of weight gain, physical activity, along with the adherence to the Mediterranean diet in its participants [13]. A systematic review had been done earlier at the start of the pandemic and concluded that this impact of COVID-19 lockdown on dietary habits was different from one community to another. Generally, the quarantine had both negative and positive effects on people’s eating habits; the positive ones included: going back to having home meals and decreasing the amount of fast food consumption, while the negative ones included: increasing the eating frequency due to quarantine and stress [23]. In other words, exploring these different variables would help plan for the medical services and community interventions in other lower-middle resourced settings. Furthermore, general lifestyle and dietary modifications could also develop the overall health status of a person, which would also prevent diseases, aside from the COVID-19-associated ones.

Based on this, this study is yet one of just a few that were done in an Egyptian setting using previously standardized study tools. Given the previously mentioned context, the current study’s objectives are to examine and evaluate changes in eating habits (i.e. changes in dietary habits during lockdown, changes in the quantity of meals and hunger sensation after pandemic in comparison to usual, and changes in the frequency of specific food items including fast food and home-made pastries and water intake). The study also evaluated the changes in general lifestyle habits (i.e. changes in physical activity and adherence after pandemic) as well as in adherence to the Mediterranean diet that occurred among a group of Egyptian adults during the COVID era.

## Methods

A cross-sectional study was done in Egypt among a group of Egyptian adults for six months from December 2020 to June 2021. It included an adult Egyptian population (≥ 18 years old) who consented to take part in and fill out the online questionnaire. The study population included adult people who could read and write and had access to the Internet. The questionnaire was uploaded on Google Forms and was shared on various social media channels (i.e. Facebook, WhatsApp, etc…). Accordingly, all Egyptians that were above 18 years old were asked to fill in the form and re-share it with other groups. In case any participant had any questions, an email address and WhatsApp number of one of the researchers were given at the end of the questionnaire.

The sample size was calculated using OpenEpi, Version3, and an open-source calculator, setting the type I error at 0.05 and confidence interval; and the width at 0.1 (with a margin of error of 5%). Results from a previous study (Renzo et al., 2020) [13] showed that the aspect of changed lifestyle habits during the COVID-19 lockdown was 37.2%. Thus, the calculation that was based on these same values produced a minimal sample of 154 adults. A 20% dropout rate was taken into account. An Arabic anonymous self-administrated online questionnaire included the following 4 sections:

### Section 1

Socio-demographic factors and special habits, such as: age, gender, residence, smoking, and sleeping habits.

### Section 2

Anthropometric data: participants were asked to report their heights in meters as well as their weights in kilograms before and after COVID-19 (1st and 2nd waves including the lockdown period).

### Section 3

A questionnaire created specifically for adults was used to examine the lifestyle using the validated Arabic version of the International Physical Activity Questionnaire Short Form (IPAQ-SF) [24]. 

As it is now the most common physical activity questionnaire, the IPAQ has two available versions: the 31-item long form (aka IPAQ-LF), and the 9-item short form (aka IPAQ-SF). The short form generally shows the activity of four specific intensity levels: the first being the vigorous-intensity activity, such as aerobics; the second being the moderate-intensity activity, such as leisure cycling; the third being walking, and the fourth being the act of sitting. Hence, using the original “last 7-day recall” form of the IPAQ-SF for the physical activity surveillance studies was suggested by the original authors.

### IPAQ Analysis

In the analysis, the frequency of moderate, vigorous, and walking exercise, as well as the duration of exercise each day, was recorded. The several activity domain categories were either handled individually to produce distinct activity patterns, or were multiplied by their value in METs and then added to reach a total figure of physical activity per week. To explain this, one MET represents the energy that is expended, while sitting and being inactive is equal to 3.5 ml/kg/min of VO2 Max. Thus, the intensity values of the MET that were used to score the IPAQ questions in this study were: vigorous (or 8 METs), moderate (or 4 METs), and walking (or 3.3 METs). Accordingly, MET-Min week-1 was calculated, and thus, categorized into low, medium, and high physical activity [24–26]. 

### Section 4

Questions to find out dietary habits.

- The first section asked about adherence to the MD, using the validated Arabic version of 14-item Mediterranean Diet Adherence Screener (MEDAS) [24]. A scored food frequency questionnaire that was previously validated was used to measure adherence to the MD. From this 14-item questionnaire, it was seen that 12 important nutrients were consumed by participants, and that two eating patterns were related to the MD. Each of these 14 items was given a score of 1 or 0 based on whether individuals adhere to each MD component (1 point) or not (0 points), The study questionnaire asked about dietary intake by serving units of olive oil, fruits or vegetables, red meat and other meat products, fish and chicken, commercial sweets, nuts and legumes, carbonated beverages, butter, and oil products.
- The final section of the questionnaire was about the changes in meal type (regarding the type of food whether healthy or not, the number of meals per day, the food cravings, home-delivered fast food intake, homemade sweets), and the amount of water intake in glasses.

### MEDAS Analysis

In the analysis, the score is between 0 and 14. The study participants were put into three groups based on their MEDAS scores: either low adherence (i.e. score 0–5), medium adherence (i.e. score 6–9), or strong adherence (i.e. score 10) to the MD [24, 27–29]. 

- As a reward or incentive for taking part in the study, some health education information was added to the questionnaire at its end. The food pyramid and advantages of routine PA were incorporated into this.

A pilot study covering 10% of the estimated sample size was conducted. It was designed to evaluate the study questionnaire’s clarity and usefulness. The final results did not include any of the pilot data.

### Data Management and Analysis

After the collected data was revised, coded, and entered on a computer, the SPSS package version 20 was then used to analyze the data. Mean, standard deviation (SD), as well as range values were done as quantitative data. As for the qualitative data, it was described using percentages and frequencies. To compare quantitative variables within the same group between two time points (before and after COVID 19), a Paired T-test was used. A Chi-square test was used to examine the relationship between the qualitative variables, a Chi-square test was used. A marginal homogeneity test was also used to compare sleeping hours categories before and after COVID-19. Moreover, a Multivariate Logistic Regression Analysis was done to estimate the adjusted odds ratios (aOR) and 95% confidence intervals (CI) to test for the association between changes in lifestyle factors and socio-demographic characteristics. Results were then considered to be statistically significant if *P*-value < 0.05. The normality of the data was examined before analysis using Histogram and Shapiro-Wilk test.

Body Mass Index (BMI) calculation was based on the following equation: BMI (kg/m^2^) = Weight (Kg)/ Height^2^ (m).

## Results

Table 1 shows that most of the study sample were females 153 (74.6%) who were living in Greater Cairo 189 (92.2%), residing in urban areas 200 (97.6%), and had a high level of education (93.2%). Around a quarter of the participants who answered the online questionnaire were not working/on pension 57 (27.8%). Moreover, about (75%) 153 participants were married.

**Table 1:** Participants’ Socio-Demographics (*n* = 205)

Character		N(%)
Sex	Male	52(25.4)
	Female	153(74.6)
Age	Mean ± SD	36.9 ± 8.02
Residence	Greater Cairo	189(92.2)
	Lower Egypt	13(6.3)
	Upper Egypt	3(1.5)
	Urban	200(97.6)
	Rural	5(2.4)
Education	Basic Secondary	7(3.4)
	Middle (Diplome/high institute)	7(3.4)
	High (University/postgraduate)	191(93.2)
Occupation	Employee/ officer	28(13.7)
	Doctor/ engineer/pharmacist/ University professor	82(40.0)
	Not working/ pension	57(27.8)
	Teacher/lawyer/accountant/ translation services	24(11.7)
	Free business	8(3.9)
Marital Status	Single	38(18.5)
	Married	153(74.6)
	Divorced/widow	14(6.8)

Table 2 shows that most of the participants were non-smokers. Around 15 participants (7%) increased the frequency of smoking while 14 (6.8%) were able to stop smoking during the COVID era. When participants were asked about the hunger sensation, 132 (64.7%) reported being more frequently hungry during the day; 52 (25.5%) remained the same; while 20 (9.8%) expressed having more frequent satiety than before the lockdown. Concerning the change in dietary habits, around 76 (37%) did not change their habits; 67 (32.7%) changed to unhealthy; while 58 (28.2%) achieved improvement to healthy styles in their dietary habits. Daily meal changes were also reported in 69 (33.6%) and 56 (27.4%) of the participants, with a decrease and increase in the amount of main meals or snacks, respectively. Fast food delivery was reported by a major percent of study subjects; 123 (60%) reported that they used to order food 1–3 times/week, and 16 (7.8%) ordered up to 7 times/week. Home-made pastries and sweets were consumed by the majority 148 (72.2%) < 3 times /week while 16 (7.8%) ate them > 5 times /week. Drinking water (≥ 8 glasses /day) was observed in only 75 (37.3%). Low adherence to the MD was found in 108 (52.7%) of the participants, while moderate and high adherence were found in 93 (45.3%) and 4 (2%), respectively. Moreover, PA clearly decreased. On the IPA questionnaire, around 118 (57.6%) of the participants scored poorly, while 125 (61%) of the participants perceived their PA activity level as reduced compared to their pre-lockdown PA.

**Table 2:** Special Habits and Lifestyle Changes during COVID-19 (*n* = 205)

Character		N (%)
Smoking Habits	Never been a smoker.	174 (84.9)
	I was a smoker but stopped during lockdown.	14 (6.8)
	I was a smoker but increased during lockdown.	15 (7.3)
	I was a non-smoker but became a smoker.	2 (1.0)
Eating Habits		
Change in Number of Meals	No change	80(39)
	Decreased number of main meals	40(19.5)
	Decreased number of snacks	29(14.1)
	Increased number of main meals	24(11.7)
	Increased number of snacks	32(15.7)
Hunger Sensation Change	No change	52 (25.5)
	Yes, I feel hungry more frequent.	132 (64.7)
	Yes, I feel satiety more frequent.	20 (9.8)
Food Delivery/Fast food	None	66 (32.2)
	1–3 times/week	123 (60.0)
	> 3 times– 7 times /week	16 (7.8)
Home-made Pastries and Sweets	< 3 times /week	148 (72.2)
	3–5 /week	41 (20.0)
	> 5 times /week	16 (7.8)
Changes in Dietary Habits During Lockdown	No change	76(37.1)
	Yes, to unhealthy	67(32.7)
	Yes, to healthy	58(28.2)
	Reduced appetite	4(2.0)
Water Intake	< 8 glasses /day	126 (62.7)
	≥ 8 glasses /day	75 (37.3)
Adherence to Physical activity (Classification based on International physical activity questionnaire)	Low	118 (57.6)
	Moderate	63 (30.7)
	High	24 (11.7)
Change in Physical Activity		
Physical activity change	No change	65 (31.7)
	Physical Activity decreased	125 (61)
	Physical Activity increased	15 (7.3)
Classification of participants according to adherence to MEDAS:	Mean ± SD	5.49 **±** 1.91
Low Adherence	0–5	108 (52.7)
Moderate Adherence	6–9	93 (45.4)
High Adherence	≥ 10	4 (2.0)

Table 3 shows that there was a statistically significant increase in the participants’ BMI (from 27.64 ± 5.26 to 28.01 ± 5.25). Furthermore, sleeping hours significantly increased during the COVID-19 era among participants; participants who used to sleep more than 9 h per night significantly increased from 3.9 to 14.3% (*p* = 0.043).

**Table 3:** Change in participants’ BMI and Sleeping hours before and during COVID-19

Change in BMI before and during COVID-19			
Character	Mean ± SD	*P* value^*^	
Before COVID-19 Pandemic	27.64 ± 5.26	0.001	
During COVID-19 Pandemic	28.01 ± 5.25		
Change in Sleeping Hours before and during COVID-19			
Character	Before COVID	During COVID	*P* value^**^
< 7 h/ d	84(41.4)	83(40.9)	0.043
7–9 h/ d	111(54.7)	91(44.8)	
> 9 h/ d	8 (3.9)	29(14.3)	

* Independent Sample T-test ** Marginal Homogeneity Test

As for Table 4, it shows factors affecting unhealthy changes in dietary habits during the COVID era. Bivariate analysis showed that changed hunger sensation and moderate MD adherence were significantly associated with unhealthy dietary habits (with *p* < 0.001 and *p* < 0.05, respectively).

**Table 4:** Factors Affecting Changed Dietary Habits to Unhealthy Style

Characteristics	Dietary Change to Unhealthy Food *n* = 143				
	Unchanged N(%)	Changed to Unhealthy N(%)	Chi square	cOR (95% CI)	aOR(95% CI)
**Age**			8.0	------	
18–25	8(66.7)	4(33.3)			Ref
> 25–35	21(40.4)	31(59.6)			2.38(0.12–47.1
> 35–45	33(55)	27(45)			4.41(0.45-43)
> 45–55	11(73.3)	4(26.7)			1.34(0.15–11.92)
> 55-	2(50)	2(50)			0.35(0.03–4.67
**Gender**			1.46	0.62(0.29–1.35)	
Male (Ref)	15(44.1)	19(55.9)			Ref
Female	61(56)	48(44)			1.76(0.62–5.1)
**Education**			2.15^#^	----------	
Basic Secondary	3(75)	1(25)			Ref
Middle	4(80)	1(20)			0.35(0.01–9.95)
High	69(51.5)	65(48.5)			0.23(0.02–3.45)
**Occupation**			1.19	1.5(0.72–3.13)	
Not Employed (Ref)	25(59.5)	17(40.7)			Ref
Employed	48(49.5)	49(50.5)			0.93(0.31–2.78)
**Marital Status**			0.22	0.84(0.4–1.74)	
Not Married (Single/Widow/Divorced)	20(50)	20(50)			Ref
Married	56(54.4)	47(45.6)			0.65(0.21–2.05)
**Residence**			0.83	1.96(0.45–8.5)	
Greater Cairo	73(54.1)	62(45.9)			Ref
Other Governorates	3(37.5)	5(62.5)			1.16(0.16–8.36)
**Changed Hunger Sensation**			35.88**	------	
No Change	34(94.4)	2(5.6)			Ref
Yes, I feel hungry more frequent.	35(36.5)	61(63.5)			29.36(6.71-130.86)*
Yes, I feel satiety more frequent.	7(63.6)	4(36.4)			6.18(0.99-38.478)
**Mediterranean Diet Adherence**			7.66^#^*	------	
Low	39(48.1)	42(51.9)			Ref
Moderate	37(63.8)	21(36.2)			2.19(0.87–5.48)
High	0(0.0)	4(100)			-----
**IPAQ score**			4.25	----	
Low	49(57.6)	36(42.4)			Ref
Moderate	21(53.8)	18(46.2)			0.19(0.04–0.87)*
High	6(31.6)	13(68.4)			0.49(0.095–2.627)

^#Fisher exact test *p<0.05 **p<0.001^

As for Table 5, it shows factors affecting reduced PA during the COVID era. Bivariate analysis showed that the changed hunger sensation was significantly associated with reduced PA (*p* < 0.001).

**Table 5:** Factors Affecting Reduction of Physical Activity

Characteristics	Physical Activity *n* = 190				
	Unchanged N(%)	Reduced N(%)	Chi square	cOR (95% CI)	aOR(95% CI)
**Age**			0.57	------	
18–25	6(33.3)	12(66.7)			Ref
> 25–35	21(33.9)	41(66.1)			1.56(0.18–13.54)
> 35–45	27(32.1)	57(67.9)			1.85(0.34–10.08)
> 45–55	7(38.9)	11(61.1)			1.89(0.36–9.97)
> 55-	3(42.9)	4(57.1)			1.62(0.24–10.73)
**Gender**			0.433	1.25(0.64–2.45)	
Male (Ref)	19(38)	31(62)			Ref
Female	46(32.9)	94(67.1)			0.59(0.27–1.28)
**Education**			1.29^#^	-------	
Basic Secondary	1(14.3)	6(85.7)			Ref
Middle	2(33.3)	4(66.7)			9.5(0.66–136.9)
High	62(35)	115(65)			1.33(0.2–8.79)
**Occupation**			1.36	1.46(0.77–2.79)	
Not Employed (Ref)	23(40.4)	34(59.6)			Ref
Employed	42(31.6)	91(68.4)			0.57(0.26–1.24)
**Marital**					
Not Married (Single/Widow/Divorced)	14(28)	36(72)	1.16	0.68(0.33–1.38)	Ref
Married	51(36.4)	89(63.6)			1.06(0.44–2.55)
**Residence**			0.006	1.043(0.34–3.19)	
Greater Cairo	60(34.3)	115(65.7)			Ref
Other Governorates	5(33.3)	10(66.7)			1.04(0.3–3.62)
**Changed Hunger Sensation**			15.26**	---------	
No change	37(50.7)	36(49.3)			Ref
Yes, I feel hungry more frequent.	12(19)	51(81)			0.27(0.7–1.02)
Yes, I feel satiety more frequent.	16(31.4)	35(68.6)			1.07(0.32–3.5)
**Mediterranean Diet Adherence**			5.02^#^	---------	
Low	29(29)	71(71)			Ref
Moderate	36(41.9)	50(58.1)			1.7(0.87–3.3)
High	0(0.0)	4(100)			---
**IPAQ Score**			3.19	----------	
Low	37(33)	75(67)			Ref
Moderate	24(41.4)	34(58.6)			0.59(0.16–2.14)
High	4(20)	16(80)			0.54(0.14–2.14)

^#Fisher exact test *p<0.05 **p<0.001^

As for Table 6, it shows factors affecting the increase in BMI during the COVID era. Bivariate analysis showed that the changed hunger sensation and reported change in PA were significantly associated with an increased BMI (*p* < 0.001). Binary logistic regression supported this result as increased PA was significantly protective from increased BMI (OR = 0.26 with 95%CI 0.11–0.66, *p* < 0.05).

**Table 6:** Factors Affecting the Change in BMI

Characteristics	Body Mass Index *n* = 150				
	**Unchanged** **N(%)**	**Increased** **N(%)**	Chi square	cOR (95% CI)	**aOR**(95% CI)
**Age**			0.83^#^	-------	
18–25	5(33.3)	10(66.7)			Ref
> 25–35	16(33.3)	32(66.7)			1.70(0.11–25.55)
> 35–45	24(34.8)	45(65.2)			1.51(0.16–14.43)
> 45–55	5(38.5)	8(61.5)			1.25(0.14–11.01)
> 55-	2(50.0)	2(50.0)			1.72(0.14–20.99)
**Gender**			0.092	0.88(0.41–1.91)	
Male (Ref)	13(33.3)	26(66.7)			Ref
Female	40(36)	71(64)			0.96(0.35–2.61)
**Education**			2.02^#^	---------	
Basic Secondary	1(16.7)	5(83.3)			Ref
Middle	0(0.0)	3(100)			13.4(0.54–334.7)
High	52(36.9)	89(63.1)			-------
**Occupation**			0.68	1.36(0.65–2.84)	
Not Employed (Ref)	17(40.5)	25(59.5)			Ref
Employed	36(33.3)	72(6 6.7)			0.78(0.28–2.12)
**Marital**			2.13	0.56(0.25–1.23)	
Not Married (Single/Widow/Divorced)	11(28.2)	31(73.8)			Ref
Married	42(38.9)	68(61.1)			1.4(0.45–4.04)
**Residence**			1.01^#^	0.52(0.14–1.89)	
Greater Cairo	48(34.3)	92(65.7)			Ref
Other Governorates	5(50.0)	5(50.0)			2.67(0.53–13.36)
**Changed Hunger Sensation**			13.47**	----------	
No change	17(56.7)	13(43.3)			Ref
Yes, I feel hungry more frequent.	29(26.6)	80(73.4)			0.79(0.12–5.22)
Yes, I feel satiety more frequent.	7(63.6)	4(36.4)			3.86(0.84–17.86)
**Changed Physical Activity**			24.26**		
No change	28(65.1)	15(34.9)			Ref
Physical activity increased	1(10)	9(90)			0.26(0.11–0.66)*
Physical activity decreased	24(24.7)	73(75.3)			6.93(0.58–85.28)
**Mediterranean Diet Adherence**			3.53^#^		
Low	26(31.7)	56(68.3)			Ref
Moderate	27(42.2)	37(57.8)			1.2(0.19–6.34)
High	0(0.0)	4(100.0)			----
**IPAQ Score**			2.54	--------	
Low	33(39.3)	51(60.7)			Ref
Moderate	17(34.0)	33(66.0)			0.46(0.091–2.32)
High	3(18.8)	13(81.3)			1.12(0.19–6.34)

^#Fisher exact test *p<0.05 **p<0.001^

Table 7 shows that regarding the use of added fat to food, only 40 (19.5%) of the participants used to consume olive oil, among whom 28 (13.5%) consumed it with the recommended daily amount (≥4 table-spoons). As for the recommended daily vegetable and fruit consumption, it was found in (118 {57.6%} and (83 {40.5%}), respectively. Moreover, weekly consumption of commercial sweets or pastries (not homemade) was found in 41 (20%) of the participants. Additionally, about 86% of the participants preferred consuming white meat like (chicken, rabbit etc…) to red meat (veal, pork etc…). Only 18 (8.8%) of the participants consumed fish or shellfish on a weekly basis.

**Table 7:** Description of Dietary habits measured by MEDAs among studied sample(*n* = 205)

Mediterranean diet adherence screener (MEDAS)	Criteria for 1 point	N(%)
1.Do you use olive oil as main culinary fat?	Yes	40 (19.5)
2. How much olive oil do you consume in a given day (including oil used for frying, salads, out-of-house meals, etc.)?	≥ 4 tbsp	28(13.5)
3. How many vegetable servings do you consume per day? (1 serving: 200 g [consider side dishes as half a serving])	≥ 2 (≥ 1 portion raw or as a salad)	118(57.6)
4. How many fruit units (including natural fruit juices) do you consume per day?	≥ 3	83(40.5)
5. How many servings of red meat, hamburger, or meat products (ham, sausage, etc.) do you consume per day? (1 serving: 100–150 g)	< 1	121(59)
6. How many servings of butter, margarine, or cream do you consume per day? (1 serving: 12 g)	< 1	82(40)
7. How many sweet or carbonated beverages do you drink per day?	< 1	44(21.5)
8. How much wine do you drink per week?	≥ 7 glasses	0(0)
9. How many servings of legumes do you consume per week? (1 serving: 150 g)	≥ 3	35(17.1)
10. How many servings of fish or shellfish do you consume per week? (1 serving: 100–150 g of fish or 4–5 units or 200 g of shellfish)	≥ 3	18(8.8)
11. How many times per week do you consume commercial sweets or pastries (not homemade), such as cakes, cookies, biscuits, or custard?	< 3	75(36.6)
12. How many servings of nuts (including peanuts) do you consume per week? (1 serving 30 g)	≥ 3	41(20)
13. Do you preferentially consume chicken, turkey, or rabbit meat instead of veal, pork, hamburger, or sausage?	Yes	176(85.9)
14. How many times per week do you consume vegetables, pasta, rice, or other dishes seasoned with sofrito (sauce made with tomato and onion, leek, or garlic and simmered with olive oil)?	≥ 2	90(43.9)

According to these aforementioned findings, the lockdown drastically changed the eating and lifestyle habits of Egyptian adults. It was seen that there were poor levels of exercise, low levels of Mediterranean Diet adherence, and high levels of eating meal delivery—up to three times a week. According to what was reported by the participants, there was a statistically significant increase in the BMI as a result of the increased appetite. Moreover, there was evidence of emotional eating, as well as a lot of baking sweets and pastries at home. However, it was seen that a few people took advantage of the pandemic and lockdown situation to change their eating habits. Accordingly, it is believed that once these habits are started, they would be hard to undo.

## Discussion

As far as is known, this study is one of the few studies that tested how the COVID-19 pandemic changed the Egyptians’ eating patterns as well as their way of life. First of all, the COVID-19 pandemic had a noticeable effect on human behavior. “Social separation” has been a common tactic used by many countries to stop COVID-19 from spreading. The lockdown had the advantage of reducing the pandemic curve as containment measures were kept in place for subsequent pandemic waves to reduce the number of cases and the strain on the healthcare system. Moreover, fears of illness and dying as well as constraints on personal freedom increased people’s stress levels and led to changes in routine activities [13]. In the current study, most of the participants were females. To explain this, the Egyptian government allowed all women with children that were under 12 years old to be exempted from work even if they were working in organizations that were abiding by the curfew hours (i.e. hospitals, or medical centers, or media outlets, or transportation of medical supplies as well as petroleum organizations). Thus, this may explain the high response rate from females.

### Dietary habits

The current study reported a changed hunger sensation and satiety; hence, there was a significant increase in the mean BMI. The amount of daily meals changed in a significant percentage of the sample. Low MD adherence was found in about half of the study participants. Moreover, both changed hunger sensation and moderate MD adherence were significantly associated with unhealthy dietary habits. These results correspond with an Italian study that was done to check the immediate effect of the COVID-19 pandemic on the people’s eating habits as well as lifestyle changes; the results reported that more than 50% of the population had a change in sense of hunger and satiety. Only 34.4% had an increased appetite to eating food; this percentage is different from that of the current study (64.7%). As for preparing and consuming homemade desserts, bread, as well as pizza, the percentage also increased. This increase is similar to that of the current study (3–5 times /week in 20% of participants) [13]. The increase in the consumption of carbohydrates (whether simple or complex) noticed in this study was obviously linked to the significant increase in the participants’ BMI (*p* = 0.001). Accordingly, the weight that is gained in the short period of any lockdown may be hard to lose for some individuals and may further lead to gaining more weight in the future if the unfavorable nutritional behaviors observed during the lockdown were not reversed [30]. 

The lockdown has affected the dietary habits of Danish adults as well. Around 28% of the respondents reported eating and snacking more with a significant degree of emotional eating. Dietary changes during that period reflected the pre-existing unhealthy eating habits that some people had. That being said, some positive health outcomes were seen in respondents with high MEDAS scores, and negative outcomes (i.e. gaining weight as well as increased intakes of carbonated beverages and pastries) were observed in respondents with low MEDAS scores [31]. 

The current study findings are different from what was observed by Renzo et al. in their Italian study; they found low, moderate, and high adherence to MD in 21.7%, 63.1%, and 15.3% of their participants, respectively [13]. Another Italian study concluded that some consumers reduced purchasing and consuming ready-to-eat foods. Also, these same consumers followed the MD carefully and ate healthier food, including adding more fresh fruits as well as vegetables in their diets [19]. This higher adherence in Italian studies may be explained by the fact that Italy is one of the origin countries that first started this important diet pattern [32]. Accordingly, additional follow-up studies are needed to examine if these habits among the Egyptian population will stick or improve with time.

Changes in dietary habits were also reported by Matsungo and Chopera in the African people of Zimbabwe. Matsungo and Chopera’s study showed that 57.8% of the participants had decreased the consumption of fruits and vegetables, while 45% showed a decrease in consuming nuts and seeds. Participants also reported a significant increase in food prices during the COVID-19 pandemic. Accordingly, this increase might undermine food security in the country. In contrast, food prices were not studied in the current study [33]. 

In the United Arab Emirates, “unhealthy” dietary patterns were also observed, with distancing from MD principles. This is added to a reported weight gain, with the majority of participants having fewer than eight cups of water per day [34]. The variation in the effect of the pandemic on the participants’ dietary habits in the above-mentioned studies may be due to the variation in the pre-pandemic habits from one country to another, as well as the availability of food items, and market accessibility. Accordingly, a change in eating habits due to the increased hunger and decreased satiety could lead to weight gain. When added to the pandemic containment measures, this might result in spending more than the usual time at home, being psychologically affected, and resulting in a consequent increase in emotional eating.

### Physical activity

As for physical activity, the study sample’s level of physical activity clearly decreased. Recreational areas and PA facilities were closed during the lockdown, followed by a partial closure during the second wave, which can explain these results. The results are similar to those of Fanelli’s research in 2021; Fanelli found that the closure of gyms and fitness facilities as well as the restrictions on visiting parks and playgrounds resulted in a decrease in PA among Italian participants [19]. Likewise, the same findings were reported in Zimbabwe, Pakistan, and Denmark [31, 33, 35]. Additionally, **Barrea et al.**, 2020, observed that patients with normal weight and Class I and II obesity showed a substantial rise in BMI values [36]. Also, **Ingram et al.,2020**, found that only 16.8% of the participants maintained the same PA level compared to the 47.4% of participants who reported a decrease in their activity [37]. 

**Barkley et al., 2020** found that the university closure increased sedentary behavior; however, the PA was decreased only in those participants who were conversely the most active before the universities were closed [38]. Closing down facilities that are designed for PA for pandemic-related reasons may disproportionately affect active individuals. This same finding was also reported in the U.S.A [39]. On the other hand, in Italy, exercise frequency increased and was done at home among those who already took part in sports, and those who used to exercise only occasionally before [13]. In Ethiopia, adopting PA increased after the pandemic in the form of performing household chores [40]. This difference in findings between studies may be attributed to the different cultures, availability of recreation places, and different methods of PA assessment.

Overall, diet and lifestyle factors, such as physical activity, are among the main risk factors -that can be modified and altered- for non-communicable diseases. These factors have thus been shown to increase the likelihood of having more severe COVID-19 negative consequences [11]. For that reason, identifying individuals most likely to engage in poor lifestyle choices during the COVID-19 pandemic is also crucial for developing interventions that are aimed at targeting these populations [34]. Further follow-up studies are required to study the long-term effects of the COVID-19 pandemic which resulted in significant disruptions to daily life.

### Smoking

In the current study, participants reported bidirectional changes in smoking habits. Similarly, in a Saudi Arabia study, about 20% of participants reported that they had already been frequent smokers; 6% reported a decline in smoking; while 4.8% reported an increased smoking rate [41]. In Italy, 3.3% of smokers managed to quit smoking, and the amount of smokers who used to smoke more than 10 cigarettes per day decreased by 0.5% [13]. The elevated risk of respiratory distress and death by COVID-19 may explain the cause of the phenomenon of decreasing or ceasing smoking [42]. Accordingly, participants who consider smoking as a stress-reliever increased their frequency of smoking, while those who perceive it as a risk factor for complications could give smoking up completely. Thus, it is seen that personal perception of danger and how to deal with it can have an impact on a smoker’s response. Follow-up studies are strongly needed to find out if those who quit or reduced smoking have consolidated their behavior or not.

### Sleeping hours

As for sleeping hours, they significantly increased among participants in the current study. Similarly, in Pakistan, most of the respondents indicated increased duration of sleep (59.5%) [35]. In the Ethiopian study, it was found that its participants had a significantly increased number of sleeping hours during the COVID-19 pandemic (with *p* < 0.0001) [40]. Also, **Ingram et al.,2020**, who assessed the change in sleep quality, reported that only 31.1% of the studied sample maintained the same sleeping pattern, while 52.4% and 16.6% showed worse and better changes, respectively [37]. 

In the U.A.E, sleep disturbances were common in 60.8% of the participants, with a major decrease in the number of participants who reported sleeping < 7 h at night, i.e. decreasing from 51.7% before the pandemic to 39% after it. An Italian study also reported a deterioration in the percentage of good sleepers after quarantine, without a significant change in the sleep duration [34, 36]. In other words, an increase in sleep duration was not necessarily accompanied by good sleep quality. Conversely, the quality of sleep was not measured in the current study. The increased sleep duration happened as a result of staying at home, nonessential businesses closing, and working online or from home rules that were applied to reduce spreading the COVID-19 infection. Enough sleep is vital for the proper functioning of the immune system as insufficient sleep may increase the susceptibility to viral infection [43, 44]. 

### Factors affecting the unhealthy change in dietary habits

Examining the factors affecting the unhealthy changes in dietary habits shows that feeling hungry more frequently and being moderately active are the main factors (*p* < 0.05). This is different from what was seen in a French study among adults NutriNet-Santé cohort. The aforementioned participants were grouped into multiple clusters. Cluster 3, for example, showed favorable nutritional changes along with an increased physical activity, compared to before [45]. This difference may be explained by the fact that they are different cultures. In the current study, this may relate to the belief that one’s body is able to manage excess energy intake if the person is in a state of being active. Another explanation is that some people tend to exaggerate their physical activity level. Significant connections were seen between an increased BMI, a change to unhealthy eating behavior due to the lockdown, and an increased hunger sensation. Moreover, a study performed in Belgium had similar results; the study found that an increase in BMI occurred because of the increase in sweet or salty snacks as well as carbonated beverages [46].

However, that being said, one of the significant characteristics of the Egyptian meals (which may have contributed to the increased BMI) is using bread as *ghomous* (means cutting bread into small pieces and used as a dip). Moreover, the Egyptian meals are mainly dependent on oil rather than on diary-divided fats. *Falafel* or *Ta’ameyya*, for example, is one of the common Egyptian traditional foods that is eaten by the majority of Egyptians on a daily basis. *Falafel* is made from mashed beans and is cooked by deep-frying in the pan [47]. For that reason, the type of meals may explain the increased BMI.

To complicate matters more, income is also a great obstacle for the Mediterranean communities to adhere to a nutritional system that is both successful and professional [48] Because there is a significant rate of poverty in Egypt, Egypt is a clear example of why its poor population would depend mainly on bread or the subsidized staple food, unwillingly resulting in a high energy content that is mainly empty calories. For that reason, Egypt’s poverty allows almost no room for having rich and diverse food or for adequate consumption of vital nutrients [49]. 

According to the WHO report that explained the non-communicable diseases’ risks of COVID-19, poor nutrition is a main risk factor for these non-communicable diseases. Consequently, poor nutrition reduces the immune system’s ability to prevent as well as recover from various infections. Moreover, consuming high levels of high-energy-dense food, like high-fats processed foods and simple sugars, may further lead to obesity compared to the consumption of low-energy dense and rich in fiber food, such as fruits and vegetables [50]. 

It was clear that the study sample embraced an obesogenic behavior during the COVID-19 era. It consisted of an unhealthy change in dietary habits; a reduced physical activity; and may have resulted in people’s their emotional stress and hence, emotional eating. For that reason, nation-wide surveys are strongly recommended to follow up these behaviors as well as follow up the non-communicable disease trend.

### Limitations of the study

The current study was limited in a few ways. Although the questionnaires were verified and useful, they were admittedly self-reported. Accordingly, self-reported questionnaires have one drawback, which is that respondents can change their responses to fit a preferred image of themselves (i.e. they can misrepresent data). As for the participants’ exact weight prior to the lockdown, recall bias may sadly exist. Another potential limit could be the convenience sampling method, which involved selecting participants via an online questionnaire and having a high percentage of sharing from females. This limits the generalizability of the results and hence, the external validity as well. In order to examine the long-term effects of the pandemic lockdown in this cultural setting, more research is thus required.

### Conclusion and recommendations

This study aimed to examine how the Egyptian population’s eating and exercise patterns changed during the COVID-19 lockdown and subsequent pandemic wave. According to the estimated sample size, a self-administered web-based questionnaire was done to gather the participants’ responses. The findings indicate that the lockdown significantly changed Egyptian adults’ eating and lifestyle patterns. A substantial portion of participants reported unfavorable changes to their eating and exercise routines. There were high levels of eating delivered food (up to three times per week) (60%); low levels of exercise (57.6%); and low Mediterranean Diet adherence (52.7%). A statistically significant increase was found in BMI which is a consequence of reduced activity and increased hunger sensation as reported. Moreover, emotional eating was also seen, as baking homemade pastries and sweets was also abundant. However, several individuals used the lockdown and pandemic situation as an opportunity to improve their eating habits. It is feared that establishing these habits would be difficult to reverse. To prevent the negative effects of such unhealthy habits (obesity, type II diabetes mellitus, cardiovascular illnesses, etc.), urgent public health policies and actions are required to guard against the consequences of such unhealthy behaviors.Various campaigns on mass and social media are also required to stress the importance of healthy dieting and maintaining physical activity. Institutional-based campaigns (i.e. in schools, universities, different organizations etc…) are encouraged as well. Engaging various stakeholders, including healthcare professionals, policymakers, community leaders, and the public, in designing and implementing these public health interventions is vital to make any initiative succeed. Additional follow-up studies are required to investigate the behavioral changes and health implications that occurred during this exceptional era.

On the other hand, and on a positive note, it was seen that some participants took the advantage of the lockdown and the worldwide pandemic and decided to: cease smoking i.e. 14 participants (6.8%), improve their physical activity levels i.e. 15 participants (7.3%), and change their dietary pattern to healthier i.e. 58 participants (28.2%). Some participants also feared the potential weight gain, so they reduced their number of snacks during the day i.e. 29 participants (14.1%).

## Data Availability

Available on reasonable request.

## Abbreviations

MD: Mediterranean Diet
MEDAS: Mediterranean Diet Adherence Screener
IPAQ-SF: International Physical Activity Questionnaire Short Form
BMI: Body Mass Index

## References

[1] Breaking.: Egypt Reports First Case of Coronavirus. Egyptian Streets. 14 February 2020. Archived from the original on 15 February 2020. Retrieved 14 February 2020.

[2] Jr RP. (2021). Policy and Institutional Responses to COVID-19 in the Middle East and North Africa: Egypt * https://www.brookings.edu/wp-content/uploads/2021/01/MENA-Covid-19-Survey-Egypt-January-28-2021-1.pdf.

[3] Library of congress Egypt.: New Measures to Combat a Possible Second Wave of COVID-19 Cases Implemented. https://www.loc.gov/item/global-legal-monitor/2020-11-18/egypt-new-measures-to-combat-a-possible-second-wave-of-covid-19-cases-implemented/.

[4] https://enterprise.press/issues/2020/03/15/schools-universities-ordered-closed-2-weeks-amid-covid-19-outbreak/enterprise.press › issues › 2020/03/15.

[5] Parmet WE, Sinha MS (2020). Covid-19 d the law and limits of quarantine. N Engl J Med.

[6] Diet WCRFAICR (2018). Nutrition, Physical Activity and Cancer: a global perspective. Continuous Update Project Expert Report.

[7] Wolfson JA, Leung CW (2020). Food insecurity and COVID-19: disparities in early effects for US adults. Nutrients.

[8] Niles M, Bertmann F, Belarmino E, Wentworth T, Biehl E, Neff R. The early food insecurity impacts of COVID-19. Nutrients. 2020; 12:2096. 10.3390/nu12072096. 10.3390/nu12072096 PMC7400862 32679788

[9] Pakravan-Charvadeh MR, Savari M, Khan HA, Gholamrezai S, Flora C. Determinants of household vulnerability to food insecurity during COVID-19 lockdown in a mid-term period in Iran - Public Health Nutr. 2021;24(7):1619-28. 10.1017/S1368980021000318. Epub 2021 Jan 26. Erratum in: Public Health Nutr. 2021;24(7):1972. 10.1017/S1368980021000318 PMC7884726 33494852

[10] Janssen M, Chang BPI, Hristov H, Pravst I, Profeta A, Millard J (2021). Changes in Food Consumption during the COVID-19 pandemic: analysis of consumer Survey Data from the First Lockdown Period in Denmark, Germany, and Slovenia. Front Nutr.

[11] Hibino S, Hayashida K (2022). Modifiable host factors for the Prevention and Treatment of COVID-19: Diet and Lifestyle/Diet and Lifestyle factors in the Prevention of COVID-19. Nutrients.

[12] Castro-Quezada I, Román-Viñas B, Serra-Majem L (2014). The Mediterranean diet and nutritional adequacy: a review. Nutrients.

[13] Renzo LD, Gualtieri P, Pivari F (2020). Eating habits and lifestyle changes during COVID-19 lockdown: an Italian survey. J Transl Med.

[14] García OP, Long KZ, Rosado JL (2009). Impact of micronutrient deficiencies on obesity. Nutr Rev.

[15] Childs CE, Calder PC, Miles EA (2019). Diet and immune function. MDPI AG: Nutrients.

[16] Rodríguez-Martín BC, Meule A (2015). Food craving: new contributions on its assessment, moderators, and consequences. Front Psychol.

[17] Yılmaz C, Gökmen V (2020). Neuroactive compounds in foods: occurrence, mechanism and potential health effects. Food Res Int.

[18] Muscogiuri G, Barrea L, Savastano S, Colao A (2020). Nutritional recommendations for CoVID-19 quarantine. Eur J Clin Nutr.

[19] Fanelli MR (2021). Changes in the Food-related Behaviour of Italian consumers during the COVID-19 pandemic. Foods.

[20] Torales J, O’Higgins M, Castaldelli-Maia JM, Ventriglio A (2020). The outbreak of COVID-19 coronavirus and its impact on global mental health. Int J Soc Psychiatry.

[21] Arnett DK, Blumenthal RS, Albert MA, Buroker AB, Goldberger ZD, Hahn EJ, Himmelfarb CD, Khera A, Lloyd-Jones D, McEvoy JW, Michos ED, Miedema MD, Muñoz D, Smith SC Jr, Virani SS, Williams KA, Sr, Yeboah J, Ziaeian B. 2019 ACC/AHA Guideline on the Primary Prevention of Cardiovascular Disease: A Report of the American College of Cardiology/American Heart Association Task Force on Clinical Practice Guidelines. Circulation. 2019;140(11):e596-e646. doi: 10.1161/CIR.0000000000000678. Epub 2019 Mar 17. Erratum in: Circulation. 2019;140(11):e649-e650. Erratum in: Circulation. 2020;141(4):e60. Erratum in: Circulation. 2020;141(16):e774.

[22] Mattioli AV, Sciomer S, Cocchi C, Maffei S, Gallina S (2020). Quarantine during COVID-19 outbreak: changes in diet and physical activity increase the risk of cardiovascular disease. Nutr Metab Cardiovasc Dis.

[23] Özlem A, Mehmet N. Eating Habits Changes During COVID-19 Pandemic Lockdown. ESTÜDAM Public Health Journal. 2020;5(COVID-19 Special Issue):169– 77. 10.35232/estudamhsd.796735.

[24] Hassan AA, Mokhtar AA, Samy NM, Mahmoud HA, Mohammed HS (2022). Prevalence of Prediabetes and its Associated risk factors among a sample of employees at Faculty of Medicine. Egypt J Occup Med.

[25] Lee PH, Macfarlane DJ, Lam TH, Stewart SM (2011). Validity of the International Physical Activity Questionnaire Short Form (IPAQ-SF): a systematic review. Int J Behav Nutr Phys Act.

[26] Helou K, El Helou N, Mahfouz M, Mahfouz Y, Salameh P, Harmouche-Karaki M. Validity and reliability of an adapted arabic version of the long international physical activity questionnaire. BMC Public Health. 2017;18(1):49. 10.1186/s12889-017-4599-7. Erratum in: BMC Public Health. 2017;17 (1):736. 10.1186/s12889-017-4599-7 PMC5525276 28738790

[27] Martínez-González MA, García-Arellano A, Toledo E, Salas-Salvadó J, Buil-Cosiales P, Corella D, Covas MI, Schröder H, Arós F, Gómez-Gracia E, Fiol M, Ruiz-Gutiérrez V, Lapetra J, Lamuela-Raventos RM, Serra-Majem L, Pintó X, Muñoz MA, Wärnberg J, Ros E, Estruch R (2012). PREDIMED study investigators. A 14-item Mediterranean diet assessment tool and obesity indexes among high-risk subjects: the PREDIMED trial. PLoS ONE.

[28] Russo GL, Siani A, Fogliano V, Geleijnse JM, Giacco R, Giampaoli S, Iacoviello L, Kromhout D, Lionetti L, Naska A, Pellegrini N, Riccardi G, Sofi F, Vitale M, Strazzullo P (2021). The Mediterranean diet from past to future: key concepts from the second Ancel Keys International Seminar. Nutr Metab Cardiovasc Dis.

[29] Haidar S, Shebly D, Doumiati S, Daouk S, El Tayara L (2016). Does Mediterranean Diet alone lower the risk of diabetes?. Elixir Nutr Dietetics.

[30] Bhutani S, Cooper JA (2020). COVID-19-Related home confinement in adults: Weight Gain risks and opportunities. Obes (Silver Spring).

[31] Giacalone D, Frøst MB, Rodríguez-Pérez C (2020). Reported changes in Dietary habits during the COVID-19 Lockdown in the Danish Population: the Danish COVIDiet study. Front Nutr.

[32] Sikalidis AK, Kelleher AH, Kristo AS. Mediterranean Diet. Encyclopedia [Internet]. 2021;1(2):371–87. 10.3390/encyclopedia1020031.

[33] Matsungo TM, Chopera P (2020). Effect of the COVID-19-induced lockdown on nutrition, health and lifestyle patterns among adults in Zimbabwe. BMJ Nutr Prev Health.

[34] Cheikh Ismail L, Osaili TM, Mohamad MN, Al Marzouqi A, Jarrar AH, Abu Jamous DO, Magriplis E, Ali HI, Al Sabbah H, Hasan H, AlMarzooqi LMR, Stojanovska L, Hashim M, Shaker Obaid RR, Saleh ST (2020). Al Dhaheri AS. Eating habits and Lifestyle during COVID-19 Lockdown in the United Arab Emirates: a cross-sectional study. Nutrients.

[35] Yasmin F, Asghar MS, Sahito AM, Savul S, Afridi MSI, Ahmed MJ, Shah SMI, Siddiqui SA, Nauman H, Khattak AK, Qazi S, Ullah I (2022). Dietary and lifestyle changes among Pakistani adults during COVID-19 pandemic: a nationwide cross-sectional analysis. J Family Med Prim Care.

[36] Barrea L, Pugliese G, Framondi L, Di Matteo R, Laudisio D, Savastano S, Colao A, Muscogiuri G (2020). Does Sars-Cov-2 threaten our dreams? Effect of quarantine on sleep quality and body mass index. J Transl Med.

[37] Ingram J, Maciejewski G, Hand CJ. Changes in Diet, Sleep, and Physical Activity Are Associated With Differences in Negative Mood During COVID-19 Lockdown. Front Psychol. 2020;11:588604. 10.3389/fpsyg.2020.588604. Erratum in: Front Psychol. 2020;11:605118. 10.3389/fpsyg.2020.588604 PMC7492645 32982903

[38] Barkley JE, Lepp A, Glickman E, Farnell G, Beiting J, Wiet R, Dowdell B (2020). The Acute effects of the COVID-19 pandemic on physical activity and sedentary behavior in University students and employees. Int J Exerc Sci.

[39] Meyer J, McDowell C, Lansing J, Brower C, Smith L, Tully M, Herring M (2020). Changes in physical activity and sedentary behavior in response to COVID-19 and their associations with Mental Health in 3052 US adults. Int J Environ Res Public Health.

[40] Kifle ZD, Woldeyohanins AE, Asmare B, Atanaw B, Mesafint T, Adugna M (2022). Assessment of lifestyle changes during coronavirus disease 2019 pandemic in Gondar town, Northwest Ethiopia. PLoS ONE.

[41] Bushnaq T, Algheshairy RM, Almujaydil MS, Malki AA, Alharbi HF, Barakat H (2022). Dietary habits and Lifestyle behaviors of Saudi residents during the COVID-19 pandemic: a cross-sectional study. Int J Environ Res Public Health.

[42] Brake SJ, Barnsley K, Lu W, McAlinden KD, Eapen MS, Sohal SS (2020). Smoking upregulates angiotensin-converting Enzyme-2 receptor: a potential adhesion site for Novel Coronavirus SARS-CoV-2 (Covid-19). J Clin Med.

[43] Krueger JM, Majde JA (2003). Humoral links between sleep and the immune system: research issues. Ann N Y Acad Sci.

[44] Toda H, Williams JA, Gulledge M, Sehgal A (2019). A sleep-inducing gene, nemuri, links sleep and immune function in Drosophila. Science.

[45] Deschasaux-Tanguy M, Druesne-Pecollo N, Esseddik Y (2021). Diet and physical activity during the coronavirus disease 2019 (COVID-19) lockdown (March-May 2020): results from the French NutriNet-Santé cohort study. Am J Clin Nutr.

[46] Drieskens S, Berger N, Vandevijvere S, Gisle L, Braekman E, Charafeddine R, De Ridder K, Demarest S (2021). Short-term impact of the COVID-19 confinement measures on health behaviours and weight gain among adults in Belgium. Arch Public Health.

[47] Hassan-Wassef H (2004). Food habits of the egyptians: newly emerging trends. East Mediterr Health J.

[48] CIHEAM/FAO (2015). Mediterranean food consumption patterns: diet, environment, society, economy and health. A White Paper Priority 5 of feeding knowledge Programme, Expo Milan 2015.

[49] Padilla M, Ahmed ZS, Wassef HH. In the Mediterranean Region: Overall Food Security in Quantitative Terms but Qualitative Insecurity. CIHEAM Analytic Note, No. 4, June 2005, 1-100. http://portail2.reseau-concept. net/Upload/ciheam/fichiers/ANP4.pdf.

[50] WHO 2020? #healthyathome. / healthy diet. https://www.who.int/campaigns/connectingthe-world-to-combat-coronavirus/healthyathome/healthyathome---healthy-diet.

